# Quality of meta-analysis in nursing fields: An exploration based on the JBI guidelines

**DOI:** 10.1371/journal.pone.0177648

**Published:** 2017-05-23

**Authors:** Yuying Hou, Jinhui Tian, Jun Zhang, Rongrong Yun, Zhigang Zhang, Kee-Hsin Chen, Caiyun Zhang, Bo Wang

**Affiliations:** 1 The First Clinical Medical College of Lanzhou University, Lanzhou, China; 2 Evidence-Based Medicine Center, Lanzhou University, Lanzhou, China; 3 School of Nursing, Gansu University of Traditional Chinese Medicine, Lanzhou, China; 4 School of Nursing, Lanzhou University, Lanzhou, China; 5 Department of Intensive Care Unit, The First Hospital of Lanzhou University, Lanzhou, China; 6 Department of Nursing, Taipei Municipal Wanfang Hospital, Taipei Medical University, Taipei, Taiwan; 7 Evidence-Based Knowledge Translation Center, Taipei Municipal Wanfang Hospital, Taipei Medical University, Taipei, Taiwan; 8 School of Nursing, Taipei Medical University, Taipei, Taiwan; 9 Department of Nursing, The First Hospital of Lanzhou University, Lanzhou, China; 10 Department of Nursing, Rehabilitation Hospital of Gansu Province, Lanzhou, China; University of Michigan, UNITED STATES

## Abstract

**Background:**

Meta-analysis is often regarded as one of the best sources of evidence for clinical nurses due to its rigorous design and scientific reflection of the true results of nursing interventions. The quality of a meta-analysis is critical to the work of clinical decision-makers. Therefore, the objective of this study was to use the JBI guidelines to summarize the quality of RCT-based meta-analyses of reports published in domestic nursing professional journals, with a view to standardizing the research process and reporting methods.

**Methods:**

We performed a comprehensive literature search for RCT-based meta-analyses published in Chinese professional nursing journals, from their inception to December 31, 2015, using bibliographic databases (e.g. CNKI, WanFang Database). March 1, 2017, supplementary search 2016 literature. Candidate reviews were assessed for inclusion by two independent reviewers using pre-specified eligibility criteria. We evaluated the quality of reporting of the included meta-analyses using the systematic review literature reporting specification of JBI. Analyses were performed using Excel and STATA 12.0 software.

**Results:**

Three hundred and twenty-two meta-analyses were included. According to the JBI guidelines, the overall quality of the meta-analysis report was poor. The quality of core journal reports and the implementation of retrieval were better than those of non-core journals. The nature of the authors and the availability of funding support had no significant impact on the quality of the meta-analyses. Multi-unit and multi-author collaboration can help improve the quality of meta-analyses with significant impact.

**Conclusion:**

The understanding and implementation of systematic evaluation and meta-analyses in domestic nursing professional journals is worthy of recognition, and there is more work that can be done to improve the quality of these reports. Systematic review / Meta-analysis (SR / MA) makers should include the findings of this study. Multi-institutional and multi-author collaborations appear to improve research capacity and provide more reliable evidence support for clinicians.

## Introduction

Evidence-based nursing is one branch of evidence-based medicine, in which caregivers conscientiously and judiciously combine scientific findings with clinical experience and patient desires in the clinical care decision-making process[1]. As the most important part of evidence-based nursing, clinical care evidence can be divided into four levels, of which, systematic reviews/ meta-analyses (SR/ MA) represent the first-level evidence, where the research design is more rigorous and can reflect the actual results of nursing intervention and are often regarded as one of the best evidence sources by clinicians[2–3].

Systematic review is a new method of literature synthesis. Systematic reviews systematically evaluate a series of published and unpublished studies on a specific problem, using rigorous principles and methods of document evaluation, selecting documents that meet quality standards and conducting qualitative or quantitative synthesis, to draw the best conclusions. Systematic evaluation can be qualitative or quantitative (i.e., a process involving meta-analysis)[4]. Although SR/ MA are one of the best sources of clinical evidence, only high-quality SR/ MA can provide a scientific basis for decisions made by clinicians, nurses and other decision makers. Poor quality SR/ MA may mislead decision-makers. Therefore, effective quality assessment is an important part of the proper use of SR/ MA, as well as caution with the use of any findings.

The Joanna Briggs Institute (JBI) is a non-profit global organization that was founded in 1996. It is the second evidence-based care center following the establishment of the Evidence-Based Nursing Center at York University. It is also the largest international organization to promote evidence-based care in the world. As with other international organizations, JBI is committed to standardizing the process of systematic reviews to enhance both quality and reliability among collaborating agencies. JBI has developed a set of theories, methods and rigorous procedures for evaluating and synthesizing different types of evidence[5] and provides an overview of JBI systems that must be reported. A review of the JBI system, which focuses on the qualitative studies of intervention outcomes, should include 18 parts with 40 items, along with corresponding flowcharts[6].

Given the usefulness of the JBI system and the importance of meta-analysis to decision-makers, the main aim of this study is to use the JBI guidelines to summarize the quality of the RCT-based meta-analyses published in domestic nursing professional journals, with a view to standardizing the research process and reporting methods. The secondary aim is to explore whether the quality of meta-analyses published in core journals and non-core journals differs, and identify factors that influence the quality of meta-analyses.

## Materials and methods

### Eligibility criteria

Before we searched the literature, nursing professional journals were identified through State Administration of Press, Publication, Radio, Film and Television of The People’s Republic of China. CNKI and Wanfang database were searched for the literatures of SR/ MA in these journals. The search period included the beginning of each journal to December 31, 2015. March 1, 2017 supplementary seach 2016 literature. Repetitive literature, review, and methodological literature, systematic reviews of etiology/ diagnostics/ methodological studies, protocol, foreign abstracts or translations of systematic or meta-analyses, non-interventional quantitative systematic review, non-RCT-based meta-analyses were excluded. Only RCT-based meta-analyses in the intervention class were included.

### Data abstraction

Two reviewers independently screened the articles based on the inclusion and exclusion criteria. Articles that did not meet the inclusion criteria in title, abstract, full text, and author information were excluded. Microsoft Office Excel 2007 software was used to establish the information extraction table, and the data was extracted independently by two reviewers. Data was collected from the included meta-analyses for study characteristics (e.g. primary author and publication year, journal type, agency, number of authors, number of outcomes, instruments used for quality assessment, funding, trial registries), retrieval information (e.g. number of bibliographic databases searched, additional search methods such as grey literature, hand searching, citation mapping) and reports on the contents for each of the various items of JBI guidelines. Prior to data extraction and quality evaluation, the use of each evaluation scale was assessed, and potential problems in data extraction and content consistency were evaluated before data extraction. The formal quality assessment process was independently completed and cross-checked by two investigators and resolved in the case of differences by a third party.

### Data synthesis and quality appraisal

Two dimensions of meta-analysis reporting and retrieval conduct were explored. For the evaluation of retrieved documents, each entry was evaluated with "yes" or "no", and the number of "yes" was counted. We used the JBI guidelines to evaluate each included review to examine the by-item quality of report. The JBI guidelines contain a total of 40 items, and the response options for each domain were “compliance”, “partial compliance”, “nonconformity”. Each included study was evaluated individually, and we counted each sufficiently reported item (answer “yes” / “compliance”). The odds ratio (OR) was used as the statistical effect and the 95% confidence interval (95% CI) was calculated, differences in the quality of meta-analyses in core and non-core journals and the factors that influence the quality of the meta-analyses were analyzed using STATA 12.0 software.

## Results

### Selection and meta-analysis samples

**Fig 1:**
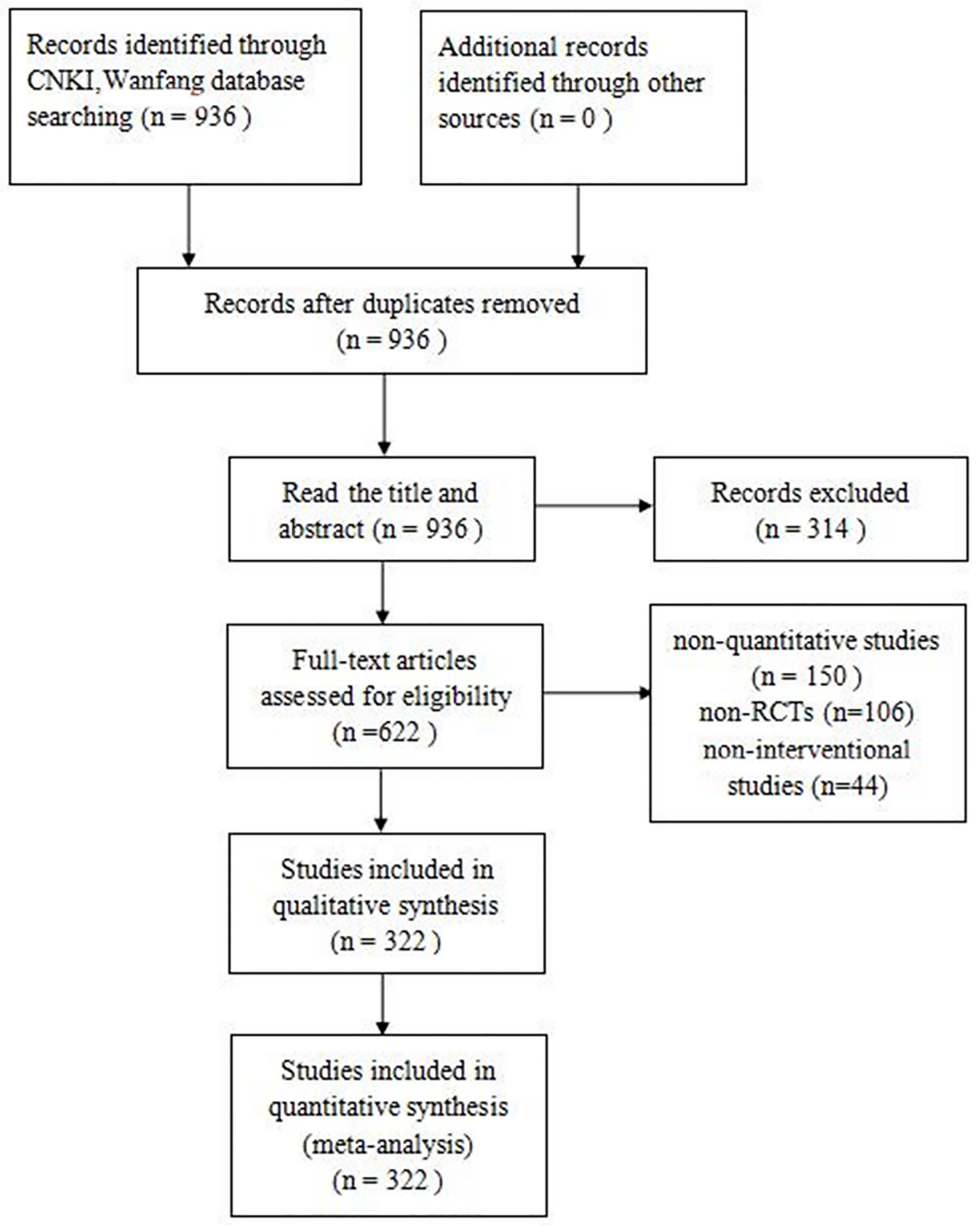
Flow chart of studies selection process in this study.

The detailed study retrieval steps according to the PRISMA statement was shown in Fig 1. In total, 936 SR/ MA were retrieved from 26 Chinese nursing journals. After reading the title and abstract, 314 studies were excluded. Following full-text review, 150 non-quantitative studies, 106 non-RCTs and 44 non-interventional studies were excluded, and a total of 322 meta-analyses were finally included.

### Characteristics of meta-analysis

Table 1 summarizes the characteristics of the included meta-analyses. The included 322 meta-analyses were from 20 of the 26 professional nursing journals in the country, including 15 core journals and 5 non-core journals. 296 articles (91.9%) were published in core journals, and 26 articles (8.1%) were published in non-core journals. Meta-analyses that met the requirements of this study were published after 2004, published in 2010 before the number of small and basically the same, after 2010, the number of published gradually increased5and faster. 83 (25.8%) of the studies were funded, but only one study was registered; 255 (79.2%) of the studies had ≥3 authors; 133 (41.3%) of the authors were from hospitals, 95 (29.5%) of the authors were from universities, 93 (28.9%) of the authors were multidisciplinary collaborations between schools and hospitals, and just 1 (0.3%) of the authors was from another institution. Of the included RCTs, the quality of the included RCTs was evaluated for 308 studies, 196 (60.9%) were evaluated using the Cochrane Handbook and 19 (5.9%) using the Jadad scale, and 16 (5%) were evaluated using the JBI standard.

**Table 1:** Characteristics of the included meta-analysis(*n* = 322).

Characteristic	Core journals(*n* = 296)	Non-core journals(*n* = 26)	All(*n* = 322)
Publish Year			
2004	1	0	1
2005	1	0	1
2007	2	0	2
2008	1	0	1
2009	1	1	2
2010	10	0	10
2011	9	0	9
2012	21	3	24
2013	27	1	28
2014	59	7	66
2015	65	14	79
2016	86	13	99
Funding			
Yes	76	7	83
No	220	19	239
Registration			
Yes	1	0	1
No	295	26	321
Number of authors			
<3	56	11	67
≥3	240	15	255
Author of the unit			
Hospital	116	17	133
University	90	5	95
Hospital and University	90	3	93
Other	0	1	1
Number of Authorities			
<3	269	26	295
≥3	27	0	27
RCT quality evaluation			
Yes	283	25	308
No	13	1	14
Quality assessment criteria	All(*n* = 283)	All(*n* = 25)	All(*n* = 308)
Cochrane	182	14	196
Jadad	13	6	19
JBI	16	0	16
Other	72	5	77

### Retrieval implementation of meta-analysis

Table 2 summarizes the retrieval implementation of the included meta-analyses. In retrieving journals from the databases, the search implementation was better in core journals than non-core journals and this difference was statistically significant (OR = 1.60, 95%CI: 1.18~2.17, *P* = 0.002). Among them, Pubmed / medline and Cochrane Library database retrieval status was significantly improved and the differences were statistically significant (Pubmed / Medline: *P* = 0.006 Cochrane Library: *P* = 0.006). There was no statistically significant difference in other database searches.

**Table 2:** Retrieval implementation of meta-analysis.

Database	Core journals(*n* = 197)		Non-core journals(*n* = 26)		OR(95%CI)
	Yes	No	Yes	No	
Pubmed/Medline	233	63	14	12	3.17(1.40,7.20)
Cochrane Library	157	139	6	20	3.76(1.47,9.64)
CENTRAL	10	286	1	25	0.87(0.11,7.11)
EMBASE	127	169	6	20	2.50(0.98,6.42)
CINAHL	16	280	2	24	0.69(0.15,3.16)
Web of Science	31	265	3	23	0.90(0.25,3.16)
PSYCHINFO	2	294	0	26	0.45(0.02,9.62)
EBSCO	28	268	0	26	5.63(0.33,94.79)
OVID	30	266	2	24	1.35(0.30,6.01)
BIOSIS	4	292	0	26	0.82(0.04,15.56)
CBM	188	108	12	14	2.03(0.91,4.55)
WanFang Database	225	71	20	6	0.95(0.37,2.46)
CNKI	261	35	24	2	0.62(0.14,2.74)
VIP	194	102	19	7	0.70(0.29,1.72)

### Reporting of meta-analysis: Compliance with the JBI guideline

Table 3 summarizes the compliance of the included meta-analysis reports with the JBI guidelines. None of the literature fully complied with the JBI guidelines. The overall completeness of the published articles in the core journals was higher than that of those published in the non-core journals and this difference was statistically significant (OR = 1.37, 95%CI:1.10~1.70, *P* = 0.005). Among these reports, the completion rate of item 11, item 19 and item 33 was obviously improved and the difference was statistically significant (item11: *P* = 0.042; item 19: *P* = 0.035; item 33: *P* = 0.017).

**Table 3:** Reporting of meta-analysis: Compliance with the JBI guideline.

Item^a^	Core journals(n = 296)		Non-core journals(n = 26)		OR(95%CI)
	Yes	No	Yes	No	
1	147	149	11	15	1.35(0.60,3.03)
2	0	296	0	26	▬
3	296	0	26	0	▬
4	296	0	26	0	▬
5	0	296	0	26	▬
6	294	2	26	0	2.22(0.10,47.51)
7	0	296	0	26	▬
	1	295	0	26	0.27(0.01,6.77)
	0	296	0	26	▬
	0	296	0	26	▬
	14	282	1	25	1.24(0.16,9.83)
8	0	296	0	26	▬
9	43	253	7	19	0.46(0.18,1.16)
10	0	296	0	26	▬
11	73	233	11	15	0.43(0.19,0.97)
12	1	295	0	26	0.27(0.01,6.77)
13	294	2	26	0	2.22(0.10,47.51)
14	294	2	26	0	2.22(0.10,47.51)
15	0	296	0	26	▬
16	296	0	26	0	▬
17	0	296	0	26	▬
18	196	100	15	11	1.44(0.64,3.25)
19	218	78	14	12	2.40(1.06,5.40)
20	211	85	18	8	1.10(0.46,2.63)
21	277	19	24	2	1.21(0.27,5.53)
22	241	55	22	4	0.80(0.26,2.41)
23	26	270	0	26	5.19(0.31,87.65)
24	216	80	15	11	1.98(0.87,4.49)
25	1	295	0	26	0.27(0.01,6.77)
26	296	0	26	0	▬
27	275	21	22	4	2.38(0.75,7.55)
28	51	245	4	22	1.14(0.38,3.46)
29	1	295	0	26	0.27(0.01,6.77)
30	216	80	15	11	1.98(0.87,4.49)
31	296	0	26	0	▬
32	113	183	11	15	0.84(0.37,1.90)
33	250	46	17	9	2.88(1.21,6.85)
34	201	95	13	13	2.12(0.94,4.74)
35	136	160	8	18	1.91(0.81,4.54)
36	11	285	0	26	2.13(0.12,37.25)
37	15	281	0	26	2.92(0.17,50.16)
38	296	0	26	0	▬
39	0	296	0	26	▬
40	296	0	26	0	▬

^a^: 1: Title 2: Reviewer 3: Author 4: Abstract 5: Background 6: Objective or Question 7: Inclusion criteria; Patient or Population; Invention; Types of study; Outcome; 8: Retrieval strategy 9: Methodological quality 10: Data collection 11: Data synthesis 12: Result 13: Conclusion 14: Key words 15: Introduction 16: Background 17:Objective or Question 18: Inclusion criteria 19: Patient or Population 20: Invention 21: Types of study 22: Outcome 23: Retrieval strategy 24: Methodological quality 25: Data collection 26: Data synthesis 27: Result 28: PRISMA flow diagram 29: Research Description (Table) 30: Methodological quality (Table) 31: Systematic review of results 32: Discussion 33: Conclusion 34: Practical Inspiration 35:Research Inspiration 36: Conflict of Interest 37: Acknowledgments 38: References 39: Appendix 40: References. (The same below).

Table 4 summarizes the compliance of the included meta-analysis reports with the JBI guidelines of each item, stratified by published year. Overall, the completeness of the items is not relevant to the year of publication. Of the 40 items, seven items (item 3, item 4, item 16, item 26, item 31, item 38, item 40) that were included in the reports were fully compliant with the JBI guidelines. All reports were completely inconsistent in the following items with the JBI guidelines: item 2, item 5, Inclusion criteria、Invention、types of study in item 7, item 8, item 10, item 15, item 17, item 39.

**Table 4:** Reporting of meta-analysis: Compliance with the JBI guideline, stratified by published year.

	Year											
Item	2004	2005	2007	2008	2009	2010	2011	2012	2013	2014	2015	2016
1	0/1^b^	0/1	2/2	0/1	1/2	4/10	5/9	15/24	15/28	40/66	37/79	39/99
2	0/1	0/1	0/2	0/1	0/2	0/10	0/9	0/24	0/28	0/66	0/79	0/99
3	1/1	1/1	2/2	1/1	1/2	10/10	9/9	24/24	28/28	66/66	79/79	99/99
4	1/1	1/1	2/2	1/1	1/2	10/10	9/9	24/24	28/28	65/66	78/79	99/99
5	0/1	0/1	0/2	0/1	0/2	0/10	0/9	0/24	0/28	0/66	0/79	0/99
6	1/1	1/1	2/2	1/1	2/2	10/10	9/9	24/24	28/28	65/66	78/79	99/99
7	0/1	0/1	0/2	0/1	0/2	0/10	0/9	0/24	0/28	0/66	0/79	0/99
	0/1	0/1	0/2	0/1	0/2	0/10	0/9	0/24	1/28	0/66	0/79	0/99
	0/1	0/1	0/2	0/1	0/2	0/10	0/9	0/24	0/28	0/66	0/79	0/99
	0/1	0/1	0/2	0/1	0/2	0/10	0/9	0/24	0/28	0/66	0/79	0/99
	0/1	0/1	1/2	0/1	0/2	2/10	0/9	1/24	2/28	3/66	6/79	0/99
8	0/1	0/1	0/2	0/1	0/2	0/10	0/9	0/24	1/28	0/66	0/79	0/99
9	1/1	1/1	0/2	0/1	0/2	1/10	1/9	2/24	2/28	8/66	11/79	22/99
10	0/1	0/1	0/2	0/1	0/2	0/10	0/9	0/24	0/28	0/66	0/79	0/99
11	0/1	0/1	0/2	0/1	0/2	0/10	0/9	0/24	0/28	0/66	1/79	83/99
12	0/1	0/1	0/2	0/1	0/2	0/10	0/9	0/24	0/28	0/66	0/79	1/99
13	1/1	1/1	2/2	1/1	2/2	10/10	9/9	24/24	28/28	65/66	78/79	99/99
14	1/1	1/1	2/2	1/1	2/2	10/10	9/9	24/24	28/28	65/66	78/79	99/99
15	0/1	0/1	0/2	0/1	0/2	0/10	0/9	0/24	0/28	0/66	0/79	0/99
16	1/1	1/1	2/2	1/1	2/2	10/10	9/9	24/24	28/28	66/66	79/79	99/99
17	0/1	0/1	0/2	0/1	0/2	0/10	0/9	0/24	0/28	0/66	0/79	0/99
18	0/1	0/1	0/2	0/1	1/2	6/10	7/9	14/24	15/28	36/66	54/79	78/99
19	0/1	0/1	1/2	1/1	1/2	6/10	8/9	18/24	22/28	53/66	70/79	52/99
20	0/1	0/1	0/2	0/1	1/2	8/10	8/9	16/24	16/28	51/66	60/79	69/99
21	0/1	0/1	2/2	0/1	2/2	10/10	9/9	21/24	24/28	63/66	74/79	96/99
22	0/1	0/1	1/2	1/1	2/2	6/10	8/9	19/24	21/28	48/66	67/79	90/99
23	0/1	0/1	0/2	0/1	0/2	2/10	0/9	3/24	2/28	1/66	9/79	9/99
24	1/1	1/1	1/2	1/1	1/2	7/10	4/9	16/24	16/28	48/66	55/79	81/99
25	0/1	0/1	0/2	0/1	0/2	0/10	0/9	0/24	0/28	0/66	1/79	0/99
26	1/1	1/1	2/2	1/1	2/2	10/10	9/9	24/24	28/28	66/66	79/79	99/99
27	1/1	1/1	1/2	1/1	1/2	9/10	7/9	21/24	27/28	60/66	70/79	98/99
28	0/1	0/1	0/2	0/1	0/2	0/10	0/9	2/24	1/28	8/66	19/79	25/99
29	0/1	0/1	0/2	0/1	0/2	0/10	0/9	0/24	0/28	0/66	1/79	0/99
30	1/1	1/1	1/2	1/1	0/2	7/10	4/9	16/24	16/28	48/66	55/79	0/99
31	1/1	1/1	2/2	1/1	2/2	10/10	9/9	24/24	28/28	66/66	79/79	99/99
32	1/1	0/1	1/2	1/1	0/2	2/10	1/9	8/24	5/28	28/66	30/79	47/99
33	0/1	0/1	1/2	1/1	2/2	9/10	6/9	22/24	24/28	52/66	67/79	83/99
34	1/1	1/1	1/2	1/1	2/2	4/10	4/9	17/24	18/28	44/66	55/79	68/99
35	0/1	0/1	0/2	1/1	0/2	0/10	2/9	8/24	7/28	27/66	25/79	74/99
36	0/1	0/1	0/2	0/1	0/2	0/10	0/9	0/24	0/28	0/66	0/79	11/99
37	0/1	0/1	0/2	0/1	0/2	0/10	0/9	0/24	0/28	2/66	2/79	11/99
38	1/1	1/1	2/2	1/1	2/2	10/10	9/9	24/24	28/28	66/66	79/79	99/99
39	0/1	0/1	0/2	0/1	0/2	0/10	0/9	0/24	0/28	0/66	0/79	0/99
40	1/1	1/1	2/2	1/1	2/2	10/10	9/9	24/24	28/28	66/66	79/79	99/99

^b^: Compliance number/ Total number

### Variables associated with JBI guidelines, stratified by funding status

Overall, there was no significant difference in the quality of the reports with or without funding (OR = 1.13, 95%CI: 0.98~1.30; *P* = 0.098), but the reporting completeness of item 18 was significantly increased in studies that were funded (item 18: OR = 1.78, 95%CI: 1.02~3.12, *P* = 0.043).

### Variables associated with JBI guidelines, stratified by author institution status

The overall quality of the JBI report from studies with multi-agency cooperation (Hospital and University) was higher than those from hospitals alone and the difference was statistically significant. The items with statistically significant differences were: item 18, item 20, item 22, item 28, item 32, item 33, item 34 and item 35. Furthermore, the overall quality of the JBI report in studies from universities was higher than those from hospitals. The items that had statistically significant differences were: item 18, item 19, Item20, item 23, item 27, Item28, Item33 and item 35. There was no statistically significant difference in other items. (Table 5)

**Table 5:** Stratified by author institution status.

	OR(95%CI)	*P*-Value
Multi-agency cooperation (Hospital and University) VS Hospital		
Overall	1.59(1.37~1.85)	0.000
Item18	1.92(1.09~3.37)	0.024
Item20	1.86(1.03~3.34)	0.039
Item22	2.88(1.34~6.18)	0.007
Item28	2.82(1.27~6.27)	0.011
Item32	1.98(1.14~3.43)	0.015
Item33	2.39(1.13~5.02)	0.022
Item34	2.61(1.44~4.73)	0.002
Item35	1.79(1.04~3.07)	0.035
University VS Hospital		
Overall	1.68(1.45~1.95)	0.000
Item18	2.11(1.20~3.72)	0.010
Item19	2.48(1.33~4.62)	0.004
Item20	2.32(1.27~4.24)	0.007
Item23	3.98(1.37~11.57)	0.011
Item27	6.48(1.45~28.91)	0.014
Item28	4.09(1.90~8.77)	0.000
Item33	2.24(1.09~4.62)	0.029
Item35	1.82(1.07~3.11)	0.028

### Variables associated with JBI guidelines, stratified by the number of authors

Overall, the JBI report quality was superior when ≥3 authors were involved, compared to when there were <3 authors and this difference was statistically significant. The completion rate of item 1, item 22, item 34 increased and the difference was statistically significant when ≥3 authors were involved.(Table 6)

**Table 6:** Stratified by the number of authors.

	OR(95%CI)	*P*-Value
≥3 authors VS <3 authors		
Overall	1.74(1.50~2.03)	0.000
Item9	4.70(1.41~15.60)	0.012
Item20	1.78(1.01~3.13)	0.046
Item28	8.53(2.02~35.96)	0.004
Item34	2.52(1.45~4.37)	0.001

### Variables associated with JBI guidelines, stratified by the number of author units

Overall, the JBI report quality was greater when ≥2 author units were involved, compared to when <2 author units were involved and this difference was statistically significant (OR = 1.27, 95%CI: 1.11~1.46, *P* = 0.001). Multi-unit cooperation led to a significant increase in the reporting completeness of item 22 and 34, with a statistically significant difference (item 22: OR = 2.27, 95%CI: 1.10~4.71, *P* = 0.027; item 34: OR = 2.12, 95%CI: 1.21~3.68, *P* = 0.008).

In summary, the quality of meta-analyses published in core journals was better than that of non-core journals. The implementation of literature searches was better in the core journals than the non-core journals. The nature of the authors also affects the quality of the article. Overall quality of the JBI report from studies with multi-agency cooperation (Hospital and University) was higher than those from hospitals alone. Furthermore, the overall quality of the JBI report from studies with universities was superior to those from hospitals. The presence or absence of funding had no significant impact on the quality of the meta-analysis reports. When the number of authors was ≥3, the quality of the meta-analyses was better than when the number of authors <3, and multi-unit cooperation (≥2) was better than a single unit.

## Discussion

RCT-based meta-analyses published in Chinese nursing journals were retrieved from a variety of sources. Since 2010, there has been a rapid increase in the number of research articles. Ninety-nine meta-analyses based on RCT were published in 2016, accounting for 30.7% of the total. From the number of authors and the author’s unit, more and more studies tended towards multi-institutional and multi-disciplinary cooperation. This trend is conducive to diversification and sustainable development.

The overall quality of the reports based on the JBI guideline in the literature was poor, and none of the studies were fully compliant with the JBI guidelines. However, the quality of articles published in core journals was significantly higher than those in non-core journals, which indicates that the efforts of domestic core journals in understanding and implementing SR / MA are worthy of recognition, but also have a lot of room for improvement. Multidisciplinary collaboration and multi-researcher have significantly improved the quality of meta-analyses, which has implications for future research and encourages multi-disciplinary collaboration to improve research quality.

The quality of the 322 included meta-analyses was variable. The major problems were: 1) the inclusion of multiple items in the abstract that did not meet the JBI guidelines (item 5, inclusion criteria, invention and types of study in item 7, 10, 12, 15), which may be related to the Chinese journals and their abstract requirements. There was a significant lack of registration number information. The registration of systematic reviews can reduce the risk of too many systematic reviews of the same topic[7,8], but also improve the transparency and credibility of any updates. 2) In the text, 91.9% of the studies did not report a complete search strategy, and the development of a reasonable and detailed search strategy can improve the recall rate and precision of the literature to ensure that the quality of the systematic evaluation, but also that the search results can be reproduced. 82.9% of the studies did not provide a PRISMA flow chart; 3) In the discussion section, only 38.5% of the studies described in detail the implications of the findings for future studies; only 0.62% of the studies address conflicts of interest, and potential stakeholder involvement could affect research design, implementation, and positive reporting of outcomes. Ignoring conflicts of interest may exaggerate the interpretation of the results, with unpredictable consequences[9,10].

In addition, the included meta-analyses also had certain positive merits: 1) 71.7% of the studies described in detail the methodological quality of the articles and improved the reliability of the results; 2) 66.5% of the studies reported the significance of research to clinical practice.

Multidisciplinary collaboration significantly improved the quality of meta-analysis reporting. The number of reports with ≥ 3 authors was significantly higher than those with <3 authors, which was in accordance with the minimum number of participants in the systematic review. The quality of the JBI report from studies with multi-agency cooperation (Hospital and University) was higher than those from hospitals alone and the difference was statistically significant. Furthermore, the overall quality of the JBI report in studies from universities was higher than those from hospitals. This may be related to the nature of the work of the authors, the degree of emphasis on scientific research, the direction of the research, but also shows that multi-agency, cross-professional cooperation is a current and future research trend.

### Limitations of this study

1) The inclusion and screening of the literature is strongly subjective, and we still cannot completely rule out missing and false detection; 2) Although the quality evaluation of this study was carried out by two independent assessors and the evaluators were trained and pre-evaluated before the evaluation, the influence of subjective factors cannot be eliminated and may affect the objectivity of the evaluation.

## Conclusions

Evaluation of 322 RCT-based meta-analyses in the domestic nursing field showed that overall reporting quality was poor based on the JBI guidelines. There were 34 of 40 items with varying degrees of missing information. In recent years, the number of meta-analyses has been increasing rapidly. In order to provide high-quality evidence support for clinicians and decision-makers, according to the shortcomings found in this study, future studies should have: 1) Strict compliance with the JBI guidelines for report writing, especially the design of the methodology should strictly abide by the relevant requirements of the JBI items to improve the strength of evidence; 2) Fellows engaged in SR / MA trained in epidemiology, statistics, and computer science to improve the ability of researchers to conduct evidence-based research, to control potential biases and to ensure reproducible and reliable results. 3) An increase in the number of multi-unit co-authors to improve the quality of the study. 4) Improving the quality of original studies, which greatly influence the quality of the evidence from the SR / MA.

## Supporting information

S1 Table PRISMA 2009 checklist. (DOC)

## References

[1] Hu Yan, Xing Weijie. Concept and Steps of evidence—based nursing[J]. *Shanghai Nursing*. 2015,15(1): 89–93.

[2] Hu Yan. “Evidence—based Nursing” and Clinical Nursing Practice[J]. *Nurs J Chin PLA*, 2002,19:3–4.

[3] Paul M, Leibovici L. Systematic review or meta-analysis? Their place in the evidence hierarchy[J]. *Clinical Microbiology Infection*.2014,20:97–100. doi: 10.1111/1469-0691.12489 24354996 10.1111/1469-0691.12489

[4] Li You-Ping, Yang Ke-Hu. Evidence—Based Medicine[M]. Beijing: People's Medical Publishing House 2014,110–111.

[5] Pearson A, Wiechula R, Court A, Lockwood C. The JBI model of evidence-based healthcare[J]. *Int J Evid Based Healthc*, 2005, 3(8): 207–215. doi: 10.1111/j.1479-6988.2005.00026.x 21631749 10.1111/j.1479-6988.2005.00026.x

[6] Sun Feng. Explanation of Medical Research Report Norms[M]. Beijing: Peking University Medical Press 2015, 265.

[7] Bagshaw SM, McAlister FA, Manns BJ, Ghali WA. Acetylcysteine in the prevention of contrast-induced nephropathy: a case study of the pitfalls in the evolution of evidence. *Arch Intern Med*, 2006, 116(2): 161–166. 10.1001/archinte.166.2.161 16432083

[8] Biondi-Zoccai GG, Lotrionte M, Abbate A, Testa L, Remigi E, Burzotta F, et al Compliance with QUOROM and quality of reporting of overlapping meta analyses on the role of acetylcysteine in the prevention of contrast associated nephropathy: Case study. *BMJ*, 2006, 322(7535), 202–209. 10.1136/bmj.38693.516782.7C PMC1352049 16415336

[9] An Ni, Xu Jun-Feng, Ge Long, Liang Li, Shi Xin-Tong, Zhou Wei-Wen, et al Reporting quality assessment of systematic reviews or meta-analysis of interventions published in Chinese Journal of Evidence-Based Pediatrics. *Chin J Evid Based Pediatr*. 2013, 8(2), 110–115.

[10] Xu Jun-Feng, An Ni, Zhou Wei-Wen, Shi Xin-Tong, Liu Yin-Chun, Liang Li, et al Methodological Quality Assessment of Systematic Reviews or Meta-Analyses of Intervention Published in the Chinese Journal of Evidence-Based Medicine. *Chin J Evid Based Pediatr*. 2013, 13(5): 605–611.

